# In Vitro Conservation through Slow Growth Storage Technique of Fruit Species: An Overview of the Last 10 Years

**DOI:** 10.3390/plants11233188

**Published:** 2022-11-22

**Authors:** Carla Benelli, Waed Tarraf, Tolga Izgu, Anna De Carlo

**Affiliations:** Institute of BioEconomy, National Research Council (CNR/IBE), Sesto Fiorentino, 50019 Florence, Italy

**Keywords:** ex situ conservation, minimal growth storage, in vitro banking, storage culture conditions, shoot culture, temperate and tropical species

## Abstract

Plant genetic resources conservation may be a potential option for the improvement of agricultural crops through modern biotechnologies, and in vitro conservation is a tool available to safeguard plant biodiversity. Ex situ conservation of plant genetic resources using the in vitro procedures is in progress in many countries. The slow growth storage (SGS) technique is a valid in vitro approach to preserve several vegetatively propagated species by controlling the growth and development of plantlets, economizing storage space and labor and reducing costs. Moreover, SGS prolongs the timing between subcultures, lowers the risk of losing germplasm through handling errors, such as contamination problems, and decreases the risk of genetic instability due to the reduction in the number of subcultures. SGS is applied by considering different factors: temperature, light or darkness conditions, medium composition, including mineral or sucrose concentrations, and the presence/absence of plant growth regulators, osmotic agents and growth inhibitors. SGS protocols for some fruit species have been well defined, others require additional research. The present review focuses on the effect of several factors that influence the SGS of in vitro shoots derived from temperate and tropical fruit species during the last ten years.

## 1. Introduction

Nowadays, plant biotechnology offers important options for the collection, molecular characterization, pathogen indexing and elimination, propagation, preservation and exchange of disease-free plant genetic resources. In particular, in vitro techniques can provide a potential contribution to overcome some of the issues related to plant genetic resources preservation [1].

Seed banking is the most efficient method of ex situ preservation [2], but it is restricted for some species that are characterized by the null/limited production of seeds, recalcitrant seed or low germination. The traditional method of preserving vegetatively propagated species is the maintenance of clonal field collections which may include a large number of accessions representing a wide range of genetic diversity [3,4]. However, the cuttings do not always suffice or do not respond adequately as propagules for propagation. Moreover, this method is costly and at constant risk of serious losses because of biotic and abiotic stresses. In the past, the Sharka virus on plums [5,6] and, recently, the development of *Xylella* emergency on olive trees [7] are examples of biotic stresses that highlight the need for complementary ex situ conservation strategies, such as slow growth storage. In vitro shoot cultures are used for the medium-term conservation of plant germplasm; indeed, well-established shoot cultures can have their growth and development in vitro slowed by modified culture conditions that affect the normal metabolism.

The technique is generally named “slow growth storage” (SGS) or “minimal growth storage” due to the use of different physical, chemical or nutritional parameters that limit the growth of the plantlets. It may also be called “cold storage” when low temperatures are applied instead of standard growth conditions.

Basically, SGS prolongs the timing between subcultures with respect to the regular intervals at 3–5 weeks depending on the species, enhancing the conservation safety as a result of fewer interferences with the culture system and minimizing the risk of contamination during the subculture process. In vitro plants of many different species may tolerate the same standard SGS conditions. However, there are most likely other species that may require species-specific conditions. Therefore, for under-researched species, each physical or chemical factor may need to be assessed.

Furthermore, SGS is applied in commercial micropropagation laboratories as a suitable strategy for short- to medium-term storage of plant materials in limited space, offering the market high-quality produce of commercial cultivars at a reduced cost.

SGS can take advantage of investigations on the effects of plant growth regulators and growth retardants, quality and quantity of light, temperature and light interactions, propagule type and growth stage. In the past, a few reviews [8,9] and several book chapters [4,10,11,12,13,14] described SGS conditions for different species, while this paper presents an overview of SGS applications for shoot conservation of temperate and tropical fruit species during the last ten years.

## 2. Factors Affecting SGS of Shoot Cultures of Temperate Fruit Crops

Several factors can interfere with the normal growth and quality of in vitro shoots, such as the temperature applied, the presence or absence of light and its intensity, the medium composition (e.g., macroelements, carbohydrates, plant growth regulators, osmotically active substances, growth retardants or antioxidant compounds) and the characteristics of the storage containers. All these factors can influence in vitro growth of plantlets at different degrees; they can also have synergetic effects. The interplay of all elements will determine the maximum conservation time in vitro, which may differ from species to species and frequently among cultivars of the same species.

In temperate fruit plants, as a general rule, a good protocol of conservation can lead to a long conservation time ranging from a few months to more than 4 years (Table 1) maintaining the viability and potentiality of shoots to regrow under standard culture conditions.

The shoot size or the number of nodes used in SGS depends on the species/genotypes and quite often also varies with the practice adopted in a specific tissue culture laboratory. Commonly, in fruit species, shoots of 2–4 cm length [14] derived from healthy in vitro cultures are important to start SGS [37]. Generally, shoots subcultured for no more than 6 cycles starting from the in vitro establishment are preferred, while shoots coming from over 12 cycles (~one year) are less suitable for SGS. Moreover, the selected shoots must be in healthy conditions [31], showing no signs of physiological disorder such as hyperhydricity, apex necrosis, chlorophyll degradation, browning or decay. For example, shoot cultures with bacterial infection or even with primary symptoms of hyperhydricity must be avoided, especially when low-temperature conditions are applied. Indeed, the thermal shock might stimulate the growth of latent bacteria, subsequently leading to substantial contamination, either during the slow growth conservation period or after regrowth and development under standard growth conditions.

The main factors determining the storage period and quality of preserved shoots will be analyzed below.

### 2.1. Temperature and Light Conditions

The most commonly used method for reducing growth in SGS is the ‘cold storage’ of shoot cultures. The incubation at a temperature lower than that required for optimum growth will reduce the metabolic activities, such as respiration, water loss, wilting and ethylene production. Reduced metabolic activities, in turn, will ensure the secure preservation of shoot cultures, resulting in the restricted growth of the plantlets [13].

Although the chosen temperature usually depends on the sensitivity of the species, it is reported that the suitable storage temperature ranges from 2 to 5 °C for temperate fruit species, while for tropical and subtropical species, from 10 to 15 °C [38].

Cold storage is often combined with the reduction of light intensity or total darkness. Most of the stored cultures are maintained under dark conditions, even though several studies have demonstrated that different combinations of photoperiod and light intensity lead to better SGS results (Table 1). The storage of shoots in total darkness is mainly used by commercial tissue culture laboratories, given the low costs required to equip a storage chamber.

Most of the species reported are stored at 4 °C (Table 1). Eleven fruit species were maintained at 4 °C in the dark for at least 12 months with the highest survival rate (100%) in *Prunus* spp. [27] and *Fragaria* spp. [21].

*Vaccinium myrtillus* shoots showed the highest survival rate (90%) and recovery rate (80%) after cold storage at 4 °C in darkness for up to 6 months [32]. Arbeloa et al. [18] conducted the same cold storage protocol (4 °C and darkness) for 18 fruit species (*Crataegus*, *Cydonia*, *Eriobotrya*, *Ficus*, *Malus*, *Prunus*, *Punica* and *Pyrus*). All species showed a survival rate of ~99% when the storage period ranged from 7 to 12 months. After 7 months of SGS, the multiplication rate (number of shoots/shoot) in *Crataegus* was 13, while in *Prunus* spp. and *Malus* it was 6 and 4, respectively, compared to other species with lower values.

Other storage temperatures are also reported, such as *Vitis* spp. stored at 10 °C [33] and 15 °C [35] for 1 year, *Arbutus unedo* and *Ceratonia sativa* at 18 °C for 6 months [15], *Citrus jambhiri* at 22 °C for 1 year [17] and *Prunus mahaleb* at 25 °C for 4 months [26].

After the storage in darkness, to retrieve the shoots and to overcome the visible elongation and etiolation of the shoots during the storage period, it is necessary to continue the subcultures under standard growth conditions [39]. In this context, different combinations of photoperiod and light intensity were more effective than total darkness when aiming at producing high-quality shoots with a fast recovery rate during the post-conservation period.

Various temperate fruit species have been investigated in storage under a combination of low temperature and low light intensity. The shoots have been stored in photoperiods ranging from 10h to 16h and light intensity from 25 to 40 µmol m^−2^ s^−1^, and the response of the species to these conditions was quite different: from 50% to 100% in terms of survival after SGS (Table 1).

In *Castanea sativa*, the effects of light and temperature were evaluated under the SGS [16]. Particularly, a dark condition was compared with a reduced light intensity (30 µmol m^−2^ s^−1^) using two storage temperatures of 8 and 4 °C. The application of a low light level and temperature of 8 °C produced positive results over long preservation periods: 82% of chestnut shoots survived after 48 months of storage. At 4 °C, the survival of shoots declined dramatically, reaching approximately 56% after 12 months, and no plants could be recovered after 24 months of storage. Higher survival rates (over 90%) were obtained with shoots of *Prunus avium* × *P. cerasus* (Gisela^®^5) after storage at 4 °C in standard growth light conditions (intensity 40 μmol m^−2^ s^−1^ and photoperiod 16 h) [19]. In another study on *Vitis vinifera*, the application of 3000 lux (55 µmol m^−2^ s^−1^) as light intensity combined with higher storage temperature (15 °C) allowed the maintenance of shoots for 12 months [35].

### 2.2. Storage Medium Composition

The components of nutrient media have a great influence on increasing the interval period between subcultures in vitro SGS. Generally, the concentration of carbohydrates, minerals, growth regulators or osmotic agents are modified in the culture medium to reduce cell division, and therefore limit the development of shoots and the formation of a callus [9].

Usually, the shoot cultures of many plants are stored in the same medium composition (macroelements, microelements and organics) used for proliferation in standard culture conditions. Among the media, the Murashige and Skoog (MS [40]) is still today the most commonly applied, although other basal micropropagation media, such as the woody plant medium (WPM [41]) and Driver and Kuniyuki walnut medium (DKW [42]), are also utilized (Table 1). These media can be applied with full or reduced strength (concentration) of their salts. In addition, specific nutrient media with special formulations are also used such as the olive medium (OM [43]) for the storage of olive shoot cultures in dark at a low temperature (4 °C) [44,45], or Knop medium [46] for the *Fragaria* in vitro storage [21].

Shoots from *Prunus*, *Punica*, *Ficus*, *Cydonia*, *Pyrus*, *Malus*, *Eriobotrya* and *Crataegus* species were successfully stored for 7 months, on MS or half-strength MS with a reduced concentration of sucrose (2%, instead of 3%) [18]. The authors noted higher multiplication rates mainly in ½MS than in full-strength MS, most likely related to the nutritive or osmotic effects of reducing sugar and mineral nutrients. Recently, the in vitro conservation of *Citrus jambhiri* cv. ‘Florida Rough’ lemon for up to 12 months was achieved using full WPM compared to other concentrations (½ and ¼ WPM) with 25 g L^−1^ of sucrose [17].

The effectiveness of plant growth regulators (PGR) supplied in culture media during in vitro preservation is widely discussed in the literature [20,29,31]. SGS makes use of media containing PGR and PGR-free media. As shown in Table 1, the best results are obtained with the presence of hormones in the media. The hormone most widely used is 6-Benzylaminopurine (BA), either alone or in combination with indole-3-butyric acid (IBA), 1-naphthaleneacetic acid (NAA) and/or gibberellic acid (GA_3_), at different concentrations ranging from 0.05 mg L^−1^ to 1.0 mg L^−1^ (Table 1). For instance, Capuana and Di Lonardo [16] investigated the presence or the absence of BA in WPM under light conditions: the survival of *Castanea sativa* shoots was 100% and 56%, with the hormone, compared to 76 and 32%, without PGR, after 4 and 12 months, respectively. In the same study, the total darkness with 0.44 µM of BA led to the highest survival rate (82%) after 48 months. In *Ziziphus jujuba* preservation, the absence of phytohormones in the growth media was optimal only for 3 months with 82.3% of survival, while for increasing the storage period to 10 months, with a survival rate of 78.6%, adding 1 mg L^−1^ BA and 0.05 mg L^−1^ IBA to the medium was required [36].

Optimizing the medium composition was a decisive factor in extending the preservation period of strawberry genotypes. The application of MS medium supplemented with 1 mg L^−1^ BA for SGS of *Fragaria × ananassa* [18] allowed the survival of only 20–32% of shoots for a duration of 7 months, while a period of 15 months was achieved on hormone-free Knop’s medium [21] and 15–18 months on hormone-free MS medium [20].

Both carbon sources and growth regulators affected slow growth storage of *Malus* spp. The most prolonged conservation duration of ≥36 months was obtained on PGR-free media containing sucrose and mannitol (2% each). SGS with media containing PGRs reduced the storage period to 12–18 months only [19]. In the same study, the reduction of nitrogen concentration (25–50%) increased the storage period. In contrast, the study by Kabylbekova et al. [23] on apple shoots has shown that the presence or absence of PGR in the medium did not affect the storage duration. MS medium supplemented with 3% sucrose was the most favorable medium to store apple shoots in vitro for 18–20 months. Furthermore, it has been shown that apple cultures can be stored in vitro for seven months with a 90% survival rate by slowing down their growth on MS medium with 1 mg L^−1^ BA and 30 g L^−1^ sucrose [19]. The in vitro conservation of *Prunus avium* shoots was investigated by Sedlak et al. [19] using the same multiplication medium, reporting 79.4% of survival after 7 months of storage without subculture. Later, Turdiyev et al. [20] in their study on SGS of cherries obtained the longest duration (30 months) when the cultures preserved on MS contained either sucrose alone (3%) or 2% combined with 2% mannitol, in the presence of PGRs. Furthermore, Turdiyev et al. [20] observed that MS medium supplemented with mannitol only reduced the SGS period of cherries to 12 months. A previous study on the response of in vitro cultures of *Z. jujuba* on half-strength MS medium supplemented with sucrose demonstrated the possibility to conserve shoots of this crop for ten months with a survival rate of 78.6%, while the absence of sucrose in the medium reduced the survival rate to 63% for the same conservation period [36].

The same authors investigated the effect of reduced sucrose and MS salts concentrations without PGRs for the in vitro preservation protocol of *Prunus mahaleb.* In ½MS media without sucrose, the shoots survival was 93.4% after only 3 months of conservation and 74.1% after 4 months [26]. The media composition in *Prunus domestica* and *Prunus cerasifera* did not affect the survival percentage during the cold preservation, even if several concentrations of sucrose, BAP and IBA were tested in MS-based media [27]. The in vitro conservation via SGS of *Prunus webbii* succeeded on the same medium used during proliferation phase, reporting a maximal survival rate after 3 months, while increasing the storage period to 6 and 10 months resulted in a significant decline with 42.6 and 15.6%, respectively [28].

Considering *Pyrus* spp. preservation, MS medium is mostly used for SGS. Lukoševıčıūtė et al. [21] reported successful storage at 4 °C for 6 months with 75–100% of shoots surviving after SGS on standard MS media plus 3% sucrose, regardless of BAP presence, versus 23–46% on MS containing 2% mannitol. Further, Sedlak et al. [19] indicated that pear shoots could be stored for 7 months on MS medium supplemented with 3% sucrose and 1 mg L^−1^ BAP, with a survival rate of 91%; Kovalchuk et al. [29] reported pear germplasm storage on MS medium without PGRs for 6–15 months, while MS medium supplemented with PGRs extended the storage period to 9–18 months. An increase in the storage duration of *Pyrus* spp. was obtained by Turdiyev et al. [20], using MS medium with a lower nitrogen content (50%), 2% sucrose and 2% mannitol in the presence of PGRs. Reed et al. [30] proved that pear shoots can be cold-stored at 1–4 °C for several years (1–4) on a specific cold storage medium of MS with half-strength nitrogen, but without PGRs. The cold storage period was less than 2 years for *P. pyrifolia* and 4 years for *P. gharbiana*, while wild *P. communis* could be stored for approximately 2 years [31]. For the cold storage of *Rubus idaeus*, MS medium supplemented with 3% sucrose, 0.5 mg L^−1^ BAP and 0.1 mg L^−1^ IBA ensured the longest conservation time; the first dead plants were noted after 15 months of storage [20]. In the same study, the storage on media with 3% sucrose without PGRs reduced the conservation period to only 12 months while adding mannitol at different concentrations, as well as its combination with sucrose, shortened the duration to 3 months with no good quality of preserved plants. Hormone-free MS medium was efficient to maintain *Vaccinium myrtillus* under slow growth storage for 6 months, recording high survival (90%) and the shoots were of good quality showing no browning symptoms [32]. In addition, SGS of *Ribes nigrum* was prolonged up to 18 months when the shoot cultures were conserved on MS medium, supplemented with PGRs (BA and IBA), 2% sucrose and 2% mannitol compared to only 12 months on the same medium but without the addition of PGRs [20].

Besides temperature and osmotic agents for short- to medium–term storage, growth retardants are also used for in vitro germplasm conservation such as abscisic acid (ABA) and Alar [47]. ABA has different physiological effects on plants, such as the inhibition of plant elongation and cell division [48] and the accumulation of proteins, leading to an increase in the resistance to stress associated with the storage conditions [49]; therefore, it is considered a plant growth inhibitor in in vitro conservation.

Indeed, 1 mg L^−1^ ABA added to the storage medium of pear genotypes increased the duration of storage for up to 36 months in ‘Mramornaya’ compared to 18 months for the control or 0.5 mg L^−1^ ABA [29]. The effect of ABA (0.5 mg L^−1^) was also evaluated by Pan et al. [33] in *Vitis heyneana* with low survival rates (26% and 23%) when the shoots were preserved at 10 °C and at 25 °C, respectively, for up to 10 months. Pre-treatments of chestnut shoots with different concentrations of ABA did not significantly influence their survival rate; an inhibitory effect on shoot proliferation was only observed at the highest ABA concentration [16].

Alar was suitable to maintain *V. vinifera* shoot tips in SGS for 12 months, with a complete regrowth ability after this storage period. The highest survival percentage (100%) was obtained with 0.4 mg L^−1^ Alar at 6 months and then declined (80 and 60%) as the duration of storage was increased to 9 and 12 months [35]. However, to improve the survival rate up to 73% after 12 months of storage, it was necessary to use MS medium containing 300 µM of ribose, without Alar.

Although the growth retardants can prolong the subculture interval, some of them may result in mutation due to their mutagenic properties and cause physiological problems if used for a longer time [50]; therefore, careful monitoring is necessary in that case.

The carbohydrates are considered a significant component for the storage medium since the use of osmotically active substances can lower the osmotic potential of the substrate, modify the growth of the shoots and affect the storage time [51].

Mainly, sucrose, mannitol, sorbitol and ribose were found to be effective in extending the storage life of in vitro grown tissues [52]. Ozudogru et al. [25] demonstrated that increasing the sucrose concentration to 45 or 60 g L^−1^ maintained the in vitro shoots of cherry rootstock ‘Gisela^®^5’ for up to 16 months, while 30 g L^−1^ sucrose-containing medium allowed the conservation of the shoot cultures for a duration of only 9 months due to the loss of material caused by hyperhydricity or decay. Monticelli et al. [22] also proved the importance of sucrose concentrations in prolonging the conservation period of apple shoots up to six months. Indeed, shoots stored on medium supplied with 130.5 mM sucrose (~44.7 g L^−1^) showed less necrosis and the highest multiplication rates after a subculture in standard growth conditions compared to those preserved on 87 mM sucrose (~30 g L^−1^). During the minimal growth storage of grapevine shoots, both osmotic agents and ribose and sucrose diversely affected the survival rate. The osmotic component showed the following effects: ribose at 300 µM (45 g L^−1^) gave the highest survival percentage (73%) after 12 months under 15 °C, followed by 53% and 47% obtained from cultures stored on media containing 200 or 100 µM (30 and 15 g L^−1^), respectively. Raising sucrose concentrations from 100 to 300 µM (from ~35 to ~103 g L^−1^) decreased the survival percentage while no survival was obtained on medium supplied with 300 µM of sucrose [35]. Further, different osmotic substances at various concentrations in ¾MS medium without growth regulators indicated a gradual decrease in the survival rate of *Vitis vinifera* shoots as the preservation period increased. After 12 months, good percentages (77.7%) of green and healthy explants were noted on media both with 3.5 or 4.5% glucose and 2.5% mannitol, but the highest percentage (88.9%) was obtained with 5.5% sorbitol [34]. The use of mannitol as an osmotic agent extended the storage time of *Vitis heyneana* under slow growth conditions, when compared with sucrose. The best survival (47.8%) was obtained at the end of 12 months of storage on MS medium supplemented with 10 g L^−1^ mannitol compared to the 46.7% reached after only 10 months of conservation on MS medium containing 40 g L^−1^ sucrose [33].

### 2.3. Containers for SGS

The type of containers used to maximize the storage duration of shoot cultures is another factor that should be considered before carrying out SGS experiments [53,54,55]. Several culture containers are available in different shapes, materials, volume and gas-permeability. In most papers reported in Table 1, glass jars or tubes were used, while in other ones it was not specified. A few studies tested different containers comparing the traditional glass jar with other types. Koç et al. [24] used the GA-7 Magenta™ box (a polypropylene container) to store *Pistacia lentiscus* shoots, while Giannì and Sottile [27] utilized the Microbox ECo 2™ (a polycarbonate container) for the conservation of two Italian plum species.

**Figure 1:**
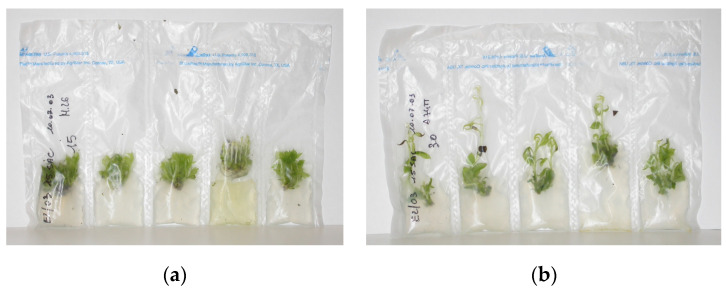
Slow growth storage of apple (**a**) and pear (**b**) shoots in Starpac Pac ™ bags (Photos by Benelli Carla).

Ozudogru et al. [25] compared the effects of gas-tight (glass jars) and gas-permeable (Star Pac™ bags) culture containers on the development of an effective in vitro SGS protocol (4 °C, darkness) for ‘Gisela^®^5’ shoots. The Star Pac™ are heat-sealable, gas-permeable plastic containers made of five cells (15 × 4 cm in size) (Figure 1). The authors evidenced an excess of CO_2_ and ethylene accumulation inside the gas-tight containers during the first weeks, referring to the physical stress of shoots as a consequence of the transfer from standard culture conditions to the conservation at low temperature and darkness. However, gas-chromatographic analysis during storage showed that the stress was quickly overcome, as both CO_2_ and ethylene concentrations were drastically reduced after 1 month of conservation. For this reason, it is more favorable to use a new generation of polystyrene bags that allow for a limited exchange of the main gases produced during tissue culture with the outside, avoiding the consistent accumulation that can be observed in traditional glass jars.

The Star Pac™ bags were also used by Turdiyev et al. [20] for the SGS of genotypes from different fruit species (apple, pear, plum, cherry, raspberry, black currant and strawberry). The authors reported that the duration of storage depended on various factors (genotype, temperature and light intensity) including the type of container, showing that plastic air-permeable containers (Star Pac™ bags) were more effective for in vitro conservation. The Star Pac™ bags were already used in previous years for the SGS of apple and pear [31,39] with satisfactory cold conservation of shoots. It is noted that Star Pac™ bags are used to maximize the space during the in vitro conservation of various woody and herbaceous plants and *Musa* collections in USDA centers in Corvallis and Mayaguez [56]. Overall, the container used for SGS should have a good air exchange, a desiccation barrier and avoid microbe diffusion; moreover, it should be of adequate size for the plants [31].

### 2.4. Genotype Effect

The performance of in vitro shoots is strongly affected by the genotype. In the literature, there are different examples where the results obtained with micropropagation varied from one plant variety to another [32,57]. For this reason, efforts should not only focus on developing appropriate genotype-specific protocols for tissue culture and micropropagation of plants, but also for SGS [54,58] to be applied on a wide range of accessions. Reed and De Noma [31] mentioned that storage duration can differ greatly among genotypes and from genus to genus. The synergy among all factors involved affects the outcome of SGS; even with an effective species-specific protocol, the response to SGS among genotypes within the same species can be quite different [33,59].

Gianní and Sottile [27] reported that four plum genotypes responded differently during SGS at 4 °C in darkness. The survival rate varied significantly among ‘Ariddu di Core’ 100%, ‘Sanacore’ 75% (*P. domestica*), ‘Marabolo’ and ‘Rapparino’ 25% (*P. cerasifera*). Moreover, they also observed that genotypes of *P. cerasifera* and *P. domestica* adapted well to in vitro culture, until the rooting stage.

In another study, Sedlak et al. [19] reported the conservation of six apple genotypes, three pear cultivars, two sweet cherry cultivars, three strawberry cultivars and two dwarf sweet cherry rootstocks. All genotypes were subjected to medium-term SGS for up to 7 months at 4 °C under light conditions. Their results showed that different fruit species and their cultivars had specific survival rates after SGS. In particular, the survival of the six apple genotypes varied between 18% (‘Tophola’) and 90% (‘Fragrance’), in the three pear cultivars from 10% (‘Elektra’) to 91% (‘Milada’) and in two sweet cherry cultivars the survival was 54.5% (‘Amid’) and 79.4% (‘Kasandra’). Even more evident is that the genotype affected the survival of the two dwarf sweet cherry rootstocks: 92% with ‘P-HL-A’ and 2% with ‘P-HL-C’.

Lukoševičiūtė et al. [21] underlined the effect of genotype under the SGS of different accessions from *F. × ananassa* (‘Venta’, ‘Melody’, ‘Elsanta’, ‘Holiday’, ‘Dangė’, ‘Nora’, ‘Nida’, ‘Jaunė’, ‘Saulenė’, ‘Catskill’, ‘Juni Morgon’, ‘Suvetar’, ‘Valotar’, ‘Vaiva’, ‘Jasna’ and KLP8), *F. moschata*, *F. vesca*, *F. virginiana*, *F. virginiana glauca* and *P. communis* (‘Oranzhevaya’, ‘Hasselpeare’, ‘Princesse Dagmara’, ‘Karalienė Jadvyga’, ‘Senryo’, ‘Muskatelka Seda’, ‘Koncentrat’ and No. 0408) and *P. pyraster*. The authors showed that the survival rate varied in a wide range depending on the genotype: in strawberry accessions from 11% (‘Suvetar’) to 100% (‘Nida’) after 15 months and in pear accessions from 75% (‘Oranzhevaya’) to 100% (‘Princesse Dagmara’) after 6 months of storage.

The impact of the cultivar on the stored plant quality and the duration of SGS was observed by Kovalchuk et al. [29] who successfully preserved the shoots of two *P. communis* cultivars, ‘Mramornaya’ and ‘Talgarskaya Krasavitsa’, at 4 °C for 18 and 12 months, respectively. Arbeloa et al. [18] indicated various multiplication rates of different genotypes/clones of *Prunus*, *Malus*, *Pyrus*, *Ficus* and *Cydonia* after 7 months of SGS at 4 °C in darkness.

## 3. Conservation in SGS of Tropical Fruit Species

Tropical germplasm resources have been reduced in their natural habitats by indiscriminate human activity, climate change and a corresponding increase in the incidence of pests, diseases and viruses. The conservation, distribution and use of the natural genetic diversity of tropical species should be considered essential, and therefore the creation of germplasm banks at national and international levels should be a priority [60]. The conservation in SGS represents a valid strategy not only for temperate species but also for tropical and subtropical species; Table 2 shows the tropical species preserved by in vitro shoot storage from 2012 to the present.

Tropical plant species are generally cold-sensitive so in many cases the conservation under SGS is not conducted at a low temperature [8]. Otherwise, physiological damages induced by cold stress referred to as chilling injury [61,62] may occur, with various changes in the metabolism, protein content, composition and functioning of the membranes.

The in vitro storage temperature depends on the cold sensitivity of the species; most of them are kept at 18–25 °C, while two species are stored at 5 °C, *Phoenix dactylifera* (date palm) and *Simmondsia chinensis* (jojoba), and one at 8 °C, *Carica pubescens* (papaja) (Table 2). In date palm, 70% of the shoots remained healthy at 5 °C in the dark after storage for 12 months on MS proliferation media without hormones [63]. Jojoba shoots were stored for 9 months at 5 °C in the dark on MS media containing a hormone (1 mg L^−1^ BA); the low temperature allowed the reduction of both the number and height of shoots and no necrosis was observed [64]. In the same study, the authors have verified the possibility to store the jojoba in standard culture conditions (25 °C and a 16 h photoperiod) using osmotic regulators in the media, such as mannitol or polyethylene glycol (PEG). The PEG addition showed a stronger growth reduction, but more shoot necrosis was observed, while the mannitol presence slightly increased the induction of new shoots. They concluded that a low temperature (5 °C) is better for the storage of healthy in vitro shoots of jojoba [64]. *Carica pubescens* was stored at 8 °C and a 16 h photoperiod for 6 months on ½MS medium with BA (2 ppm), showing a 90% survival rate and 100% of regrowth on recovery medium [65].

For the other tropical species listed in Table 2, the temperature was not modified with respect to the standard growth conditions during micropropagation, but changes concerned the medium composition to effectively reduce the shoot growth rate and, thus, the period between subcultures, without compromising their quality and health.

Recently, de Oliviera and Aloufa [66] tested the effect of osmotic compounds added to the culture medium to slow the growth of *Hancornia speciosa* shoots maintained at 25 °C (30 μmol m^−2^ s^−1^, 16 h photoperiod). Different concentrations of sucrose and sorbitol were tested for various storage times. Sorbitol showed a more pronounced growth-reducing effect than sucrose. The combination of 15 g L^−1^ sucrose with 5 g L^−1^ sorbitol gave the best result (95% survival), allowing the conservation of the shoots for 4 months. In contrast, higher concentrations of sucrose (30 g L^−1^) and sorbitol (10 or 20 g L^−1^) showed toxic and stressful effects on shoot survival with thin stems, reduced or absent leaves, high oxidation incidence and greater callus formation at the base of the explants [66].

Mannitol was effective in the conservation of taro (*Colocasia esculenta* var. *globulifera*); after 24 months, 80% of the shoots survived with a concentration of 4% mannitol in the culture medium [67]. Lower concentrations of mannitol (2%) allowed for the conservation of up to 6 months, while by increasing the concentration to 6%, the storage period fell to 2 months. 

The application of ABA (3 mg L^−1^) in the storage medium for the preservation of *Vanilla planifolia* resulted in reduced shoot growth and allowed a storage period of 6 [68] or 4 months [69], with a survival rate around 90%.

An in vitro collection of 66 pineapple accessions was successfully stored for 10 years at 21 °C, with a lower light intensity (20 μmol m^−2^ s^−1^) and a shorter photoperiod (12 h), with a transfer of stored shoot cultures on fresh medium every 24 months [70]. Although all shoots were viable after this long period of storage, in post-conservation the capacity of the recovery and propagation potential was genotype-dependent. A few accessions required only one subculture (45 days) for a full restoration as new cultures, but other accessions needed 3–5 subcultures.

Bananas and plantains are among the most important tropical fruit crops worldwide. Local and global efforts for the ex situ preservation of banana germplasm are massive. For example, at the International Transit Centre (ITC) in Leuven (Belgium), the in vitro SGS is achieved under a combination of low temperature (16 °C) and limited light (25 μmol m^−2^ s^−1^) [71,72]. Therefore, this method is appropriate to decrease the subculture frequency, ranging between 3 and 22 months, depending on the genomic group and in vitro browning reaction [71]. In particular, in the ITC, the AAA Mutika Lujugira bananas and AAB non-plantain bananas can be stored for up to 390 days, while the wild bananas (*M. acuminata* and *M. balbisiana*) require a subculture after 275 days. Other accessions (Lady Finger-Pome, AAB) achieved 615 days of storage compared to 60 days for SF215, a AA *M. acuminata* sp. *banksii* derivative [73,74].

Kanchanapoom and Promsorn [75] reported that sucrose (1%) was a suitable carbon source for storing shoots of *Musa balbisiana* ‘Kluai Hin’ (BBB group) at 25 °C and a 16 h photoperiod for 6 months. Other sources of carbohydrates (glucose and sorbitol) or higher concentrations of sucrose (3 and 5%) did not positively affect the storage duration of the cultures, and also conservation under dark conditions did not improve the regrowth capacity of the shoots.

Eight media with MS or ½MS containing different concentrations of hormones (BA and IAA) and sucrose (20, 30, and 60 g L^−1^) were tested for the preservation of three cultivars of banana at 26 or 18 °C [76]. After 5 months, the highest survival percentage (100%) was obtained with 2.25 mg L^−1^ BA, 0.175 mg L^−1^ IAA and 30 g L^−1^ sucrose at 18 °C. The full or half concentration of the mineral salts did not affect the conservation of the three cultivars.

To reduce the development of banana shoots, it was possible to apply growth retardants such as ABA, maleic hydrazide and paclobutrazol (PBZ) [77,78]. In particular, PBZ plays a key role in the inhibition of cell elongation and internodes by affecting the biosynthesis of gibberellins and increasing ABA and chlorophyll activity [79,80]. In the banana variant ‘Kepo’, the addition of PBZ at 2.5 or 5.0 ppm was effective to slow the growth of plantlet height, number of leaves and ratio of leaf length to leaf width, allowing in vitro shoot storage for 6 months at 18–22 °C. No physiological damage or loss of morphogenetic ability of the shoots were observed during the medium-term storage [81].

An innovative study evaluated the effect of different spectra of light on the conservation of the banana ‘Prata Catarina’ group AAB under SGS [72]. Plantlets maintained with a photoperiod of 14 h (25 °C) were illuminated with red and blue light (combined or alone) or with white light. Photosynthetic photon flux density (PPFD) for all treatments was set at 50 μmol m^−2^ s^−1^. Plantlets grown under the blue spectrum showed a positive response to SGS for about 5 months; the blue light reduced photosynthetic activity and consequently, induced a lower metabolic activity during the storage. To date, few studies have been carried out on the influence of light on in vitro conservation, thus it is desirable to increase the attention to this important parameter. For example, in banana accessions where wide differences in storage time conservation are recorded (ranging from 3 to 22 months) [71], the authors suggest the investigation of different light spectra to promote the storage period.

**Table 2:** Tropical fruit shoots conservation in SGS from 2012 to present. Culture conditions and best results are reported for each species (terminology and values are the same as mentioned by the authors).

Species	Medium	Temperature (°C)	Light Condition	Storage Time (months)	Survival (%)	References
*Ananas comosus*	½MS, 30 g L^−1^ sucrose	21	12 h, 20 μmol m^−2^ s^−1^	24 *	100	[70]
*Carica pubescens*	½MS, 2 ppm BA	8	16 h, 18 watts	6	90	[65]
*Colocasia esculenta*	MS, 4% mannitol	NR	16 h	24	80	[67]
*Hancornia speciosa*	MS, 15 g L^−1^ sucrose, 5 g L^−1^ sorbitol	25	16 h, 30 μmol m^−2^ s^−1^	4	95	[66]
*Musa* spp.	MS, 30 g L^−1^ sucrose	25	14 h, blue light (450–465 nm) 50 μmol m^−2^ s^−1^	3.5	100	[72]
	½MS or MS, 2.25 mg L^−1^ BAP, 0.175 mg L^−1^ IAA, 30 g L^−1^ sucrose	18	NR	5	100	[76]
	MS, 4% mannitol	23	12 h	16	NR	[55]
*Musa acuminata* × *balbisiana*	MS, 2.25 mg L^−1^ BAP, 0.175 mg L^−1^ IAA, 3% sucrose, 2.5 and 5 ppm PBZ	18-22	Natural light	6	NR	[81]
*Musa balbisiana*	MS, 1% sucrose	25	16 h, 20 μmol m^−2^ s^−1^	6	25	[75]
*Phoenix dactylifera*	MS, 10 mg L^−1^ 2,4-D, 3 mg L^−1^ 2iP, 6 mg L^−1^ ABA, 102 g L^−1^ sucrose, 3 g L^−1^ AC	15	Darkness	12	91.8	[82]
	MS	5	Darkness	12	70	[63]
*Simmondsia chinensis*	MS, 1 mg L^−1^ BA	5	Darkness	9	NR	[64]
*Vanilla planifolia*	MS, 2 mg L^−1^ BA, 3 mg L^−1^ ABA	24	16 h, 40–50 μmol m^−2^ s^−1^	6	90	[68]
	MS, 3 mg L^−1^ ABA	22	16 h, 50 μmol m^−2^ s^−1^	4	93.3	[69]

* Every 24 months the shoot cultures have been renewed for a period of 10 years. NR: not reported. MS, Murashige and Skoog medium; AC, activated charcoal; ABA, abscisic acid; BA, 6-Benzyladenine; BAP, 6-Benzylaminopurine; IAA, indole-3-acetic acid; 2,4-D, 2,4-Dichlorophenoxyacetic acid; 2iP, 6-(γ,γ-Dimethylallylamino)purine; PBZ, paclobutrazol.

Further, in tropical species, the genotype effect was documented. Several responses in shoot, leaf and root formation after 6 months of in vitro preservation were noted in four banana accessions (‘Valery’, ‘Dwarf Cavendish’, ‘Pelipita-Colombia’ and ‘Pelipita-Costa Rica’) [55]. Similar behavior was indicated by Zainy et al. [76] for three banana cultivars (C1-Pisange, C2-Brazillian and C3-William) preserved for 5 months; the application of various media and temperatures gave variability in the shoot growth of all cultivars as a result of genotype effect. In pineapple, da Silva [70] found differences in the micropropagation potential among 66 accessions after a long conservation period (10 years) under SGS, related to the response of each variety/accession.

## 4. Genetic Stability

Despite that in vitro conservation techniques were optimized for several plant species, genetic stability is considered an important aspect in SGS protocols of germplasm because in vitro conditions may cause somaclonal variation and epigenetic changes [69,83].

In vitro culture conditions, the long exposure of plant material to cytokinin, can increase the probability of somaclonal variations. Kamińska et al. [84] reported that slow growth storage and further regrowth on MS medium supplemented with BA may cause abnormalities with changes in the amount of DNA in the cells. Unbalanced concentrations of auxins and cytokinins and supra-optimal levels of growth regulators have been linked with somaclonal variation. However, the effect of the type and concentration of plant growth regulators on the incidence of somaclonal variation in different plant species remains a topic of debate [85].

Moreover, excessive generation of reactive oxygen species (ROS) in explants subjected to cold stress can also lead to somaclonal variation due to DNA damage. Indeed, in cold storage, the growth of explants is slowed down but prolonged exposure to low temperatures may cause stress, especially to thermophilic species [86].

In any case, SGS is considered as an effective approach to maintaining genetic resources in in vitro gene banks [12], and the analysis of the genetic stability of the in vitro preserved material can be a step to detecting the changes that may arise altering the genetic homogeneity of the germplasm [87].

The genetic investigation was applied by molecular markers such as random amplification of polymorphic DNA (RAPD), sequence-related amplified polymorphism (SRAP) [88], simple-sequence repeat (SSR), inter-simple sequence repeat (ISSR) [89,90], amplified fragment length polymorphism (AFLP) and start codon targeted (SCOT) [24,69,70,91].

The genetic stability, using ISSR markers, of 66 pineapple accessions after micropropagation and SGS for 10 years was reported by da Silva et al. [70]. No somaclonal variation was recorded in most of the accessions; only two accessions of *A. comosus* var. *bracteaus* showed genetic instability. Therefore, a subculture interval of 24 months is recommended by the authors for pineapple shoots kept under in vitro conservation.

Bautista-Aguilar et al. [69] demonstrated high genetic stability of *Vanilla* preserved for 4 months using ISSR and SSR markers with a low percentage of polymorphism detected in different accessions of *V. planifolia* (SSR 0%, ISSR 2%) and in *V. insignis* (SSR 0%, ISSR 0%).

Koç et al. [24] reported that *Pistacia lentiscus* adapted to in vitro cold storage and showed some genetic variations by AFLP after 6 months of storage at 4 °C. According to the results, genetic similarity values of the non-preserved and cold-stored plantlets ranged from 0.66 to 0.84, with a mean of 0.74. Thus, in vitro propagation and especially cold storage of *P. lentiscus* may be affected by transposons activation, which could cause genetic instability.

## 5. In Vitro Banking Strategy

Several countries have built up national germplasm banks for the conservation, utilization and distribution of plant material. Germplasm conservation needs to be established for preserving high-quality plant genetic sources and to set a database of available genetic material that would facilitate the knowledge and the research. Various institutions and centers in the world have produced important applicative impact with the establishment of in vitro banks, mainly of shoot cultures maintained in SGS, spread across 15 countries of 6 continents, with a conservation policy especially directed to the most strategically important plants for human nutrition. Today, over 37,000 accessions are preserved by means of SGS of shoot cultures [92]. Only three species represent over 90% of this germplasm: potato (*Solanum* spp.), with almost 19,000 accessions conserved in SGS at 6–10 °C as shoot or microtuber cultures; cassava (*Manihot esculenta*), with over 9400 accessions; and sweet potato (*Ipomea batatas*) with over 5300 accessions mainly preserved as shoot cultures at room temperature with the use of osmotically active compounds (mannitol, sorbitol and sucrose) in the storage medium [37].

Among the fruit species, the *Musa* species has the largest gene bank established in Belgium at the International Musa Germplasm Transit Centre (ITC) with ˃1600 accessions sourced from different national and regional field collections in 38 countries; these accessions are ex situ conserved under minimal growth conditions to reduce the growth rate of the plant tissues and the frequency of subculturing cycles [71]. Additionally, the Tropical Agriculture Research Station (USDA-ARS TARS) in Mayagüez, Puerto Rico, has been maintaining 164 accessions of *Musa* spp. in field collection and in in vitro storage [55].

A complete listing of worldwide fruit germplasm related to in vitro collections is difficult to find because the germplasm is often stored in in vitro conditions as collections for study and research purposes. Generally, there is a need to introduce new strategies for managing in vitro collections as a routine, as well as an appropriate collecting process. Such procedures should be designed specifically for each species and variety [93] involving the introduction, evaluation, characterization and distribution of high-quality germplasm and its preservation for long-term availability.

Particularly, the measures implemented should be complying with safety and ethical authority regulations and they should ensure: (i) healthy material: contamination-free material, (ii) authenticity: correct identification and (iii) stability: trueness-to-type. Good laboratory practices with careful application of aseptic techniques, clear and accurate documentation and the correct procedures that decrease the risks of genetic variation are all necessary activities to ensure the in vitro storage of plant material. In order to avoid somaclonal variation in SGS, the use of shoots and apices as an explant source and the reduction of the number of subcultures can be recommended.

SGS can be a useful tool to continue the conservation of plant diversity as mentioned by Target 8 of the Global Strategy for Plant Conservation (GSPC): ‘At least 75 percent of threatened plant species in ex situ collections, preferably in the country of origin, and at least 20 percent available for recovery and restoration programmes’ [94]. Indeed, SGS allows the appropriate storage of stock material to be used for restoration and provides a method for propagating plants to reduce the pressure on wild populations.

For long term conservation, many accessions can be preserved using advanced tools such as cryopreservation. Cryopreservation is the storage of biological material at an ultra-low temperature of −196 °C in liquid nitrogen, and it can be considered a safety backup method for field collections to reduce the loss of plant germplasm [95]. Currently, it involves over 10,000 accessions preserved in cryo-banks established in different countries for cassava, potato, banana, apple, pear, coffee, mulberry and garlic [71,96].

## 6. Conclusions and Perspectives

**Figure 2:**
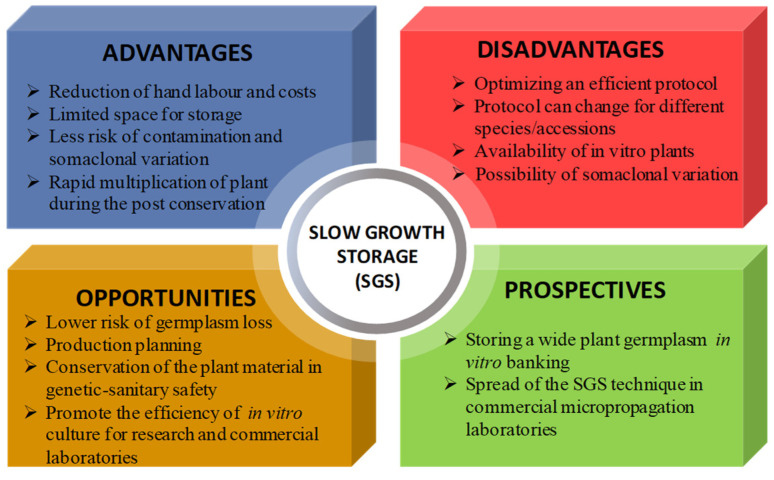
In vitro slow growth storage (SGS) technique as a competitive tool to make the plant conservation dynamic and applicable in present and future.

Ex situ plant conservation depends on the species, the methods employed and the desired storage time, and these occurrences are associated with costs, risks and scientific challenges. In vitro conservation allows for the preservation of plant genetic resources, disease-free planting material, material to be provided to growers all year round and control of the genetic fidelity verified with molecular analysis, at the same time. Particularly, SGS, with less frequency of subcultures, lowers the risk of losing germplasm through handling errors, such as contamination problems, and decreases the risks of genetic instability, manual labor, and the space needed for germplasm conservation. Thus, considering all this, SGS can also be an efficient way to reduce production costs (Figure 2).

It is evident that the simplest and most successful slow growth strategies involve temperature and light limitation as reported in this review.

SGS protocols for some fruit species require additional research, but for others are well-defined. A bottleneck in the application of slow growth is the adaptation of a generic protocol to every accession of a large and various plant genebank; the possibility of one common protocol applicable as best practice across all plants is limited mainly due to variable species and genotype responses. A standard protocol can be applied to diverse genotypes, although minor improvements may be required for outlying, low response performers. Therefore, it might be more practical to develop a number of protocols as standard operating procedures which can be validated for different accessions across different genebanks [14]. For this reason, it is important to support cooperation through knowledge sharing on the best practices developed in the plant research community (public institutions, universities and private laboratories) and active genebanks.

Overall, for in vitro storage significant precautions should be taken to use healthy plants, e.g., applying tests for virus-free material, especially for vulnerable species before initiating SGS. Further research needs to improve the in vitro conservation of plant germplasm collection and to enlarge the applications and the prospects of the SGS technique (Figure 2).

## Figures and Tables

**Table 1:** Shoot conservation of temperate fruit crops in SGS from 2012 to present. Culture conditions and best results are reported for each species (terminology and values are the same as mentioned by the authors).

Species	Medium	Temperature (°C)	Light Condition	Storage Time (Months)	Survival (%)	References
*Arbutus unedo*	MS, 1 mg L^−1^ zeatin	18	16 h, 30 μE s^−1^ m^−2^	6	80	[15]
*Castanea sativa*	WPM, 0.44 μM BA, 30 g L^−1^ sucrose	8	16 h, 30 μM m^−2^ s^−1^	48	82	[16]
*Ceratonia siliqua*	MS, 0.1 mg L^−1^ BA	18	16 h, 30 μE s^−1^ m^−2^	6	100	[15]
*Citrus jambhiri*	WPM, 25 g L^−1^ sucrose	22	12 h, 20 μmol m^−2^ s^−1^	12	NR	[17]
*Crataegus monogyna*	½MS, 5 mM BA, 0.5 mM IBA, 20 g L^−1^ sucrose	4	Darkness	7–12	99	[18]
*Cydonia oblonga*	½MS, 5 mM BA, 0.5 mM IBA, 20 g L^−1^ sucrose	4	Darkness	7–12	99	[18]
*Eriobotrya japonica*	½MS, 5 mM BA, 0.5 mM IBA, 20 g L^−1^ sucrose	4	Darkness	7–12	99	[18]
*Ficus carica*	½MS, 5 mM BA, 0.5 mM IBA, 20 g L^−1^ sucrose	4	Darkness	7–12	99	[18]
*Fragaria* x *ananassa*	MS, 1 mg L^−1^ BA, 30 g L^−1^ sucrose	4	16 h, 40 μmol m^−2^ s^−1^	7	32	[19]
*Fragaria* spp.	MS	4	10 h, 10 μmol m^−2^ s^−1^	15–18	NR	[20]
	Knop medium	4	Darkness	15	100	[21]
*Malus domestica*	½MS, 5 mM BA, 0.5 mM IBA, 20 g L^−1^ sucrose	4	Darkness	7–12	99	[18]
	MS, 1 mg L^−1^ BA, 30 g L^−1^ sucrose	4	16 h, 40 μmol m^−2^ s^−1^	7	90	[19]
	MS, 0.44 μM BA, 130.5 mM sucrose	4	Darkness	6	NR	[22]
*Malus* spp.	MS (25–50% NO_3_), 2% sucrose + 2% mannitol	4	10 h, 10 μmol m^−2^ s^−1^	≥36	NR	[20]
	MS, 3% sucrose	4	10 h, 15 μmol m^−2^ s^−1^	18–20	NR	[23]
*Pistacia lentiscus*	MS, 1 mg L^−1^ BA, 3% sucrose	4	Darkness	12	NR	[24]
*Prunus avium*	MS, 1 mg L^−1^ BA, 30 g L^−1^ sucrose	4	16 h, 40 μmol m^−2^ s^−1^	7	79.4	[19]
*Prunus avium* × *P. cerasus*	MS, 1 mg L^−1^ BA, 30 g L^−1^ sucrose	4	16 h, 40 μmol m^−2^ s^−1^	7	92	[19]
	DKW, 0.5 mg L^−1^ BA, 45 or 60 g L^−1^ sucrose	4	Darkness	16	NR	[25]
*Prunus mahaleb*	½MS media without sucrose	25	16 h, 43.4 μmol m^−2^ s^−1^	4	74.1	[26]
*Prunus* spp.	½MS, 5 mM BA, 0.5 mM IBA, 20 g L^−1^ sucrose	4	Darkness	7–12	98.6	[18]
	MS, 0.5 mg L^−1^ BA, 0.1 mg L^−1^ IBA, 2% sucrose + 2% mannitol	4	10 h, 10 μmol m^−2^ s^−1^	30	NR	[20]
	MS, 2.2 μM BA, 0.49 μM IBA, 20 g L^−1^ sucrose	4	Darkness	12	100	[27]
*Prunus webbii*	MS, 0.7 mg L^−1^ BA, 0.01 mg L^−1^ NAA, 0.1 mg L^−1^ GA_3_, 3% sucrose	4	Darkness	6	42.6	[28]
*Punica granatum*	½MS, 5 mM BA, 0.5 mM IBA, 20 g L^−1^ sucrose	4	Darkness	7–12	99	[18]
*Pyrus communis*	½MS, 5 mM BA, 0.5 mM IBA, 20 g L^−1^ sucrose	4	Darkness	7–12	99	[18]
	MS, 1 mg L^−1^ BA, 30 g L^−1^ sucrose	4	16 h, 40 μmol m^−2^ s^−1^	7	91	[19]
*Pyrus* spp.	MS, 30 g L^−1^ sucrose	4	Darkness	6	100	[21]
	MS, 3% sucrose, 0.5 mg L^−1^ BA, 0.1 mg L^−1^ IBA/without PGRs	4	10 h, 7 μmol m^−2^ s^−1^	18/15	NR	[29]
	MS (25% NO_3_), 2% sucrose, 2% mannitol	4	10 h, 10 μmol m^−2^ s^−1^	36	NR	[20]
	½MS nitrogen	1-4	12 h, (10–20 µE m^−2^ s^−1^)/dark	12–48	NR	[30]
	MS	4	12 h, 10 µE m^−2^ s^−1^	48	NR	[31]
*Ribes nigrum*	MS, 0.5 mg L^−1^ BA, 0.1 mg L^−1^ IBA, 2% sucrose + 2% mannitol	4	10 h, 10 μmol m^−2^ s^−1^	18	NR	[20]
*Rubus* spp.	MS, 0.5 mg L^−1^ BA, 0.1 mg L^−1^ IBA, 3% sucrose	4	10 h, 10 μmol m^−2^ s^−1^	15	NR	[20]
*Vaccinium myrtillus*	MS, 30 g L^−1^ sucrose	4	Darkness	6	90	[32]
*Vitis heyneana*	MS, 0.05 mg L^−1^ IBA, 0.1 mg L^−1^ IAA, 0.5 mg L^−1^ ABA, 10 g L^−1^ mannitol	10	16 h, 40 μmol m^−2^ s^−1^	12	47.8	[33]
*Vitis vinifera*	¾ MS, 5.5% sorbitol	5	Darkness	12	88.9	[34]
	MS, 300 µM ribose	15	16 h, 3000 Lux	12	73	[35]
*Ziziphus jujuba*	MS, 1 mg L^−1^ BA, 0.05 mg L^−1^ IBA, 3% sucrose	4	Darkness	10	78.6	[36]

NR: not reported. MS, Murashige and Skoog medium; WPM, woody plant medium; DKW, Driver and Kuniyuki walnut medium; ABA, abscisic acid; BA, 6-Benzyladenine; GA_3_, gibberellic acid; IAA, indole-3-acetic acid; IBA, indole-3-butyric acid; NAA, 1-Naphthaleneacetic acid; PGRs, plant growth regulators.

## Data Availability

The data presented are available in all the publications cited in this review.
